# Cytomegalovirus antibodies in dried blood spots: a minimally invasive method for assessing stress, immune function, and aging

**DOI:** 10.1186/1742-4933-8-3

**Published:** 2011-01-13

**Authors:** Jennifer B Dowd, Allison E Aiello, Laura Chyu, Yuan-yen Huang, Thomas W McDade

**Affiliations:** 1 Epidemiology and Biostatistics, Hunter College, CUNY School of Public Health, 425 E. 25th St., New York, NY 10010, USA; 2 CUNY Institute for Demographic Research (CIDR), One Bernard Baruch Way, New York, NY 10010, USA; 3 Department of Epidemiology, School of Public Health, University of Michigan, 1415 Washington Heights, 3rd Floor Tower, Ann Arbor, MI 48109, USA; 4 Center for Social Epidemiology and Population Health, University of Michigan, 1415 Washington Heights, 3rd Floor Tower, Ann Arbor, MI 48109, USA; 5 Cells to Society (C2S): The Center on Social Disparities and Health, Institute for Policy Research, Northwestern University, 2040 Sheridan Road, Evanston, IL 60208, USA; 6 Department of Anthropology, Northwestern University, 1810 Hinman Avenue, Evanston, IL 60208, USA

## Abstract

**Background:**

Cytomegalovirus (CMV) is a prevalent herpesvirus with links to both stress and aging. This paper describes and validates a minimally invasive method for assessing antibodies against CMV in finger stick whole blood spot samples for use as an indirect marker of an aspect of cell-mediated immunity.

**Results:**

Analysis of CMV in dried blood spot samples (DBS) was based on modifications of a commercially available protocol for quantifying CMV antibodies in serum or plasma. The method was evaluated through analysis of precision, reliability, linearity, and correlation between matched serum and DBS samples collected from 75 volunteers. Correlation between DBS and plasma values was linear and high (Pearson correlation *R *= .96), and precision, reliability, and linearity of the DBS assay were within acceptable ranges.

**Conclusions:**

The validity of a DBS assay for CMV antibodies will enable its inclusion in population-based surveys and other studies collecting DBS samples in non-clinical settings, increasing scientific understanding of the interaction of social and biological stress and immune function.

## Background

Immune system decline is one of the hallmarks of aging. Age-associated alterations in systemic immunity, referred to as 'immunosenescence', are thought to contribute to the increased incidence and severity of infectious disease in older persons, as well as decreased response to vaccination [1,2]. Increasing evidence suggests that the persistent herpesvirus cytomegalovirus (CMV) plays an important role in human immunosenescence, meriting special attention in studies designed to understand stress, immune function, and health[3]. This paper describes and validates a minimally invasive method for assessing antibodies against CMV in dried whole blood spot samples. The collection of dried blood spots (DBS)-drops of whole blood on filter paper from a single finger prick-has recently become a popular minimally invasive technique for collecting biomarkers in population surveys [4]. Since most standard laboratory protocols require serum or plasma, assay protocols must be developed specifically for DBS and validated for accuracy, precision, reliability, and limits of detection. This validation of a dried-blood spot assay for CMV antibodies will facilitate its broad inclusion in population-based surveys and other studies collecting biomarkers in non-clinical settings, increasing scientific understanding of the interaction of social and biological stress and immune function.

### The importance of cytomegalovirus (CMV) as a marker of immune function and aging

CMV is a ubiquitous herpesvirus that is most often asymptomatic and acquired early in life, with most populations reaching over 70% seroprevalence for those over age 60 [5,6]. Once acquired, CMV remains latent in the host for life, with containment of the virus becoming an immune system priority. Accumulating evidence suggests that CMV may play a direct role in immune system aging, and CMV infection has even been called the "driving force" behind age-associated alterations to the T cell immune system[3]. Higher CMV antibody levels in conjunction with CMV DNA shedding in urine indicating subclinical reactivation have been found in old compared to young subjects infected with CMV[7]. Aging populations experience increased CMV specific CD8+ T-cell accumulation and a reduction in naïve T cells, potentially reducing the availability of CD8+ T-cell carrying receptors that are specific for pathogens or foreign antigens other than CMV[8]. This CMV-specific CD8+ T cell accumulation may play a role in the decreased ability of the elderly to resist new infections, though recent rodent evidence suggests that the "immunological space" may be more flexible than previously believed[9]. Recent findings have shown that latent CMV infection in the elderly is an important component of a set of immunological parameters designated the "immunological risk phenotype"[3]. This set of immunological markers-including latent CMV infection, high CD8 cells, low CD4 cell percentages, poor T-cell proliferation-is predictive of mortality among healthy elderly individuals [3,10]. A large fraction of the available adaptive immune resource is focused on CMV, as high as 10-30% of all CD4 cells and 50% of all CD8 cells in elderly individuals[11,12]. CMV IgG antibody titers have been found to increase linearly with CMV viral load in leukocytes, suggesting they are a good indicator of host immune response to viral replication[13,14].

Increased anti-CMV IgG antibodies have been associated with higher levels of the inflammatory cytokines TNF-**α **and IL-6 along with reduced immune response to influenza vaccination among both young and older individuals [15]. CMV-specific CD8 T cells also have the ability to produce IFN-**γ**[16]. Thus, an accumulation of CMV specific CD8 T cells may lead to an increase in several circulating inflammatory cytokines in the elderly, including TNF-**α**, IFN-**γ**, and IL-6. An increase in chronic peripheral cytokine concentration and reduced repertoire of T cells is likely to influence pathophysiological changes associated with chronic health conditions and mortality. In epidemiological studies, CMV antibodies have been linked to inflammatory processes, cardiovascular disease, frailty, cognitive decline, and mortality [17-21].

### Stress and Immune function

Adequate cell-mediated immunity is important for maintaining persistent infections such as cytomegalovirus (CMV), Epstein-Barr virus (EBV) or herpes simplex virus type 1 (HSV-1) in a latent state. Stress-related down regulation of cellular immunity can allow a herpesvirus to reactivate, releasing viral antigens into circulation. In turn, levels of antibodies to CMV, EBV, and HSV-1 provide an indirect measure of cell-mediated immune function. Findings from the psychoneuroimmunology literature support a strong and consistent relationship between stress and increased antibody response to herpesviruses [22]. Specifically, increases in herpesvirus antibody titers have been linked to academic stress in medical students and military cadets [23,24], caregiving for a family member with Alzheimer's disease [25], involvement in a poor quality marriage [22], traumatic life events [26,27], as well as the psychological traits of loneliness, defensiveness, and anxiety [28,29]. Specific to CMV, antibodies have been shown to increase in response academic stress in several student populations[24,29], and the stress of spaceflight for astronauts[30]. Increased CMV antibody titers have been associated with depression and anxiety in older adults[31]. Increased severity of parental psychiatric symptoms has also been associated with an increase in the percentage of CD4 and CD8 cells associated with immune control over CMV in school-aged children[32]. Increased replication of CMV and antibody titer has been observed in vitro and in vivo with administration of hydrocortisone [33-35], as well from stress-related catecholamine response[36].

### Dried-Blood Spot Methods for Integrating Biomarkers into Population-Based Research

The inclusion of biomarkers in social surveys has greatly expanded in recent years[37]. Dried blood spots have been incorporated into a number of major studies both in the U.S. and internationally, including the Health and Retirement Study (HRS), Los Angeles Family and Neighborhood Study (L.A.FANS), National Longitudinal Study of Adolescent Health (Add Health), National Social Life, Health, and Aging Study (NSHAP), and the Mexican Family Life Survey (MFLS). The addition of biomarkers to such community and population-based surveys highlights efforts to understand the biological pathways through which social and economic factors shape population health. Historically, these surveys have relied primarily on health data from self-reports or from vital records. The addition of biomarkers has allowed investigation of physiological processes that may be below the threshold of perception to the individual but nonetheless important predictors of future morbidity and mortality. The development of minimally invasive methods for sample collection also facilitates the inclusion of biomarkers into community-based research among a wider range of diverse populations.

DBS samples offer several advantages for collection of biomarkers, including access to physiological information that would not otherwise be attainable in a non-clinical setting and a relatively non-invasive sample collection. Furthermore, unlike plasma or serum, DBS samples do not need to be centrifuged, separated, or immediately frozen following collection, providing considerable flexibility for collection and transport. DBS samples also remain stable in laboratory freezers for long periods of time and can be analyzed at a later date as new biomarkers of interest emerge[4].

Procedures for sample collection and processing are relatively straightforward and have been described in detail[4]. Briefly, the participant's finger is cleaned with isopropyl alcohol and then pricked with a sterile, disposable lancet similar to those used for blood glucose monitoring. The first drop of blood is wiped away with gauze, and subsequent blood drops are applied to filter paper. The samples are allowed to dry from four hours to overnight, at which point they can be stacked and stored in re-sealable plastic bags. The filter paper matrix stabilizes most analytes in dried blood spots for extended periods of time, but the rate of degradation varies by analyte. Before analysis, the DBS sample must first be brought into solution. A standard hole punch is typically used to cut discs of whole blood of uniform size. One or more discs are placed into an elution buffer for a fixed amount of time, reconstituting the DBS as hemolyzed liquid whole blood, which can then be used in assays much as plasma or serum would be.

Among herpesviruses, dried-blood spot assays have currently only been validated for Epstein-Barr virus (EBV) antibodies[4,26]. While EBV has the advantage of a higher seroprevalence at younger ages[38], CMV may be a more useful measure for middle-aged and older populations because of its links to aging and its seroprevalence of over 80% among U.S adults 60 and over[39]. Compared to EBV, research has shown that CMV seems to be dominant in causing the clonal expansions of dysregulated CD8 cells that are a crucial component of immunosenescence[3]. Thus while antibodies to EBV can provide an indirect marker of cell-mediated immune function, antibody levels to CMV can provide both a marker of stress-related changes in immune function as well as a potentially clinically relevant marker of aging and immune decline. A broadly available dried blood spot assay for CMV antibody levels would facilitate research on many fronts for understanding the psychological and social factors that accelerate aging.

## Results

### Assay protocol

A commercially available enzyme immunoassay kit for measuring CMV IgG antibodies in serum or plasma (Diamedix Corporation, No.720-320, Miami, FL) was selected as the assay platform for the DBS protocol. The day before an assay was to be performed, DBS samples were removed from the freezer, and a small hole punch was used to remove one 3.2-mm diameter disk of whole blood. The disk was transferred with tweezers to a labeled 12 × 75 mm glass tube, and 250 μL of diluent buffer (supplied with the kit) were added. The sample was covered and incubated overnight at 4°C. The following day 100 uL of whole blood eluate were transferred to microtiter plate wells in duplicate. For calibration, the kit includes three levels of anti-CMV antibody in stabilized human sera, and 100 uL of each was added to the plate in duplicate. Wells were pre-coated with purified CMV antigen, and anti-CMV IgG antibody in the sample was bound to the plate during incubation. Following a wash step, anti-human IgG conjugated with horseradish peroxidase was added to bind to the antibody-antigen complex. Excess conjugate was washed away and chromogenic substrate was added to catalyze color development directly proportional to the amount of anti-CMV IgG antibody in the sample. Absorbance at 450 nm was obtained with a plate reader spectrophotometer (BioTek ELx 808, Winooski, VT) for the samples and for the calibrators.

Three calibrators were provided by the manufacturer pre-diluted, with known concentrations of CMV antibody reported in ELISA units: 0, 10, and 160 EU/mL. Absorbance values from the calibrators were used to generate a best-fit linear standard curve, and CMV antibody concentrations for each sample were calculated based on each sample's absorbance value. It is important to note that the calibrators are serum-based, while we are using whole blood eluates as our sample. Therefore we do not expect results from matched serum and DBS samples to be identical, although we do expect a highly correlated, linear relationship between the two sets of results. The regression equation characterizing this relationship can be used to calculate serum-equivalent values based on DBS results.

### Assay validation

Assay precision, or intra-assay variation, was assessed with 10 determinations of four control samples across the assay range, all analyzed in a single run. Reliability, or between-assay variation, was assessed by assaying four control samples in duplicate across five different days. For both intra- and inter-assay variation, the percent coefficient of variation was calculated for each control sample (%CV = 100 × SD/mean). Assay linearity was assessed by serially diluting three control samples 1:2, 1:4, and 1:8 in diluent buffer after elution. The ratio of observed to expected values was calculated for each dilution as a measure of assay accuracy across the assay range.

The correlation between serum and DBS results was evaluated using paired samples collected from 75 volunteers at the University of Michigan. The study was approved by the Institutional Review Boards of the University of Michigan and Hunter College, City University of New York. A trained phlebotomist first cleaned the participant's finger with isopropyl alcohol and then pricked with a sterile, disposable lancet. The first drop of blood was wiped away with gauze, and subsequent blood drops were applied to filter paper. The samples were allowed to dry overnight and were subsequently stored at -20°C in re-sealable plastic bags with desiccant. Venipuncture collection into 7.0 ml BD Vacutainer^® ^glass serum tubes occurred immediately after blood spot collection. Serum was removed, aliquotted, and stored at -80°C. Samples were transported by automobile on dry ice from the University of Michigan to the Laboratory for Human Biology at Northwestern University for analysis. DBS samples obtained by finger-prick were analyzed according to the protocol above. Serum samples were analyzed according to procedures specified by the kit for measuring CMV IgG antibodies in serum or plasma (Diamedix Corporation, No.720-320, Miami, FL).

**Figure 1:**
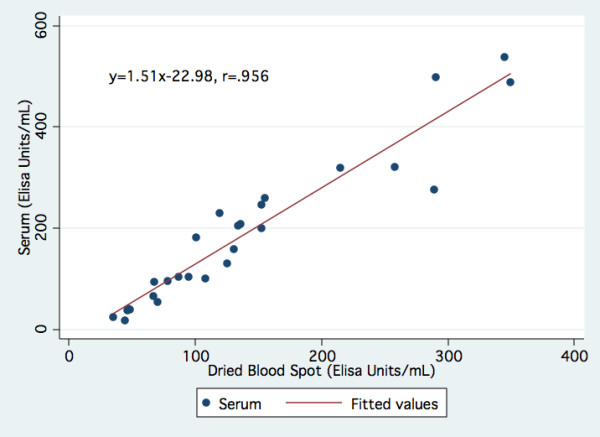
Correlation between Matched Dried Blood Spot and Serum Samples, CMV IgG

Correlation and linear regression analyses were used to evaluate the relationship between anti-CMV antibody values derived from DBS and serum samples. In addition, Bland-Altman plots were used to uncover evidence of bias or inconsistent variability across the range of measurement[40]. Seropositivity of serum samples was determined according to test kit specifications, with < 8.0 EU/ml = seronegative, 8.0-9.9 = equivocal, and ≥ 10 = seropositive. The corresponding seropositivity cut-off for DBS samples was determined based on the matched serum seropositivity results. One extremely high seropositive value (serum = 1806 EU/mL, DBS = 1040 EU/mL) was excluded from the regression calculation for Figure 1.

Correlation between blood spot and plasma values among positive serum samples was linear and high (Pearson correlation *R *= .96) (Figure 1). There were no equivocal samples among the sera, allowing definitive seropositivity determination that was then compared to DBS results. There was some overlap in positive and negative DBS samples between EUs of 35 and 55, reducing the sensitivity and specificity of the DBS seropositivity cut-off relative to the serum assay. Table 1 shows DBS sensitivity and specificity values at different DBS cut-off values. For the purposes of measuring continuous CMV antibody level in middle-aged and older populations where the majority of individuals are already seropositive, precise ascertainment of dichotomous seropositivity is less crucial and investigators may choose a lower, more lenient cut-off to reduce the number of false-negative individuals. For investigators more concerned with seropositivity as an outcome itself, a higher, more conservative cut-off can be used to help reduce the number of false-positives.

**Table 1:** DBS Seropositivity Sensitivity and Specificity

Cut-Off (EU/mL)	Sensitivity	Specificity
35	100.0%	80.9%
46	92.9%	93.6%
48.5	85.7%	95.7%
51.5	82.1%	97.9%
56	82.1%	100.0%

*Sensitvity *= % of positive serum samples testing positive with DBS

*Specificity *= % of negative serum samples testing negative with DBS

### Precision and Reliability

As seen in Table 2, precision and reliability were excellent in the middle control ranges. More variability was seen in the negative sample, as well as in the highest control sample, with 11% intra-assay variability and 10% inter-assay variability, respectively.

**Table 2:** CMV Antibody Assay Precision and Reliability

	Intra-Assay		Inter-Assay	
Control Level	(N = 10)		(N = 5)	
	
	Mean ± SD	%CV	Mean ± SD	%CV
Neg	25.6 ± 2.6	10.01	25.5 ± 3.8	15.16
Low-Pos	37.3 ± 2.4	6.4	37.6 ± 3.2	8.49
Mid-Pos	63.8 ± 7.4	11.53	67.7 ± 3.9	5.7
High-Pos	117.3 ± 13.1	11.13	135.4 ± 14.0	10.37

Mean antibody titers are presented in standard ELISA Units/mL.

### Linearity

To evaluate linearity, three high samples were serially diluted: 1:1; 1:2; 1:4; and 1:8. Mean recovery (100 × observed/expected) was 104.8%, and ranged from 98% to 112%, indicating a high degree of linearity across the assay range.

## Discussion

Results of our assay validation demonstrate that determination of CMV antibody titers using dried blood spots is feasible and provides a sensitive, precise, and reliable measure. The use of DBS methodology in population-level research has many advantages compared to venipuncture including low cost and ease of sample collection, storage, and transportation, facilitating collection in settings outside of the clinic or laboratory. Respondent burden is also minimized, increasing the potential for more frequent collection. These advantages broaden the possibility for inclusion of such markers in large, representative samples to overcome the limitations of smaller clinical studies.

Given CMV's established links to both aging and stress, the inclusion of CMV antibody levels in DBS assays from population surveys can be an important tool for elucidating how psychological and social factors translate into health outcomes over the life span. Prior to this study, of the various herpesviruses that have been linked to stress. a validated DBS assay was available only for EBV[26]. While EBV has been used effectively as marker of cell-mediated immunity related to stress, CMV may prove to be a more valuable marker for looking at both stress and immune system aging. Research has found that EBV and CMV infections induce quantitatively and qualitatively different CD8 T-cell responses in advanced aging, with EBV chronic infection kept under control by a limited and stable number of circulating CD8+ CD28+ T cells compared to CMV which seems to dominate clonal expansion[41].

Many of the biological changes that occur in response to chronic stress appear to parallel those observed in aging. Stress and aging are further linked through life changes such as bereavement and caregiving. While stress is known to elicit a variety of biological responses that can contribute to the development of disease, the specific mechanisms linking stressors to aging are not well understood. Periodic subclinical reactivation of CMV due to stress may contribute to an increase in CMV-specific T cells over time, accelerating immunosenescence[7].

The role of stress in explaining socioeconomic differences in health has been explored using neuroendocrine markers of stress such as cortisol, but the nature of cortisol measurement has contributed to the inconsistent results of these studies[42]. Antibody levels to CMV provide a promising alternative to neuroendocrine measures for understanding the dynamics of social factors, stress, and health. At the population level, CMV seroprevalence is higher in children and adults with lower socioeconomic status (SES) and non-white race/ethnicity in the U.S.[43], and socioeconomic differences in antibody response to CMV among seropositive individuals have also been identified[44]. Increased immune vigilance over CMV may take a toll on these disadvantaged groups via earlier onset of immunosenescence, as well as increased levels of inflammation and inflammatory-related chronic diseases. The addition of CMV antibodies to population-based studies of aging would allow exploration of novel hypotheses linking stress, immune function, and health outcomes at older ages.

## Abbreviations

CMV: (cytomegalovirus); DBS: (dried blood spot); IgG: (immunoglobulin G); TNF-α: (tumor necrosis factor-alpha); IL-6: (interleukin-6); IFN-γ: (interferon-gamma); HSV-1: (herpes simplex virus type 1); EBV: (Epstein-Barr virus); EU: (elisa units)

## Conflicts of interests

The authors declare that they have no competing interests.

## Authors' information

JD received her Ph.D. from Princeton University in August 2004, where she specialized in Economics and Biodemography, with a focus on socioeconomic inequalities in health. From 2006-2008, JD was a Robert Wood Johnson Health & Society Scholar in the Department of Epidemiology at the University of Michigan. She is currently Assistant Professor of Epidemiology and Biostatistics at Hunter College, City University of New York, and Faculty Associate at the CUNY Institute for Demographic Research. Her work examines how the social environment influences the immune system, with a focus on stress, chronic infections, and immune function.

AA has a PhD in Epidemiology, from Columbia University and an MS in Environmental Health Microbiology from the University of North Carolina-Chapel Hill, School of Public Health. AA is currently an Associate Professor of Epidemiology at the University of Michigan, in the Center for Social Epidemiology and Population Health. AA's research includes the use of cohort data and laboratory testing of biological samples for latent infections and inflammation. AA's research focuses on the social and immunological determinants of age-related health declines and the application of psychoneuroimmunological methods in population based data. She has experience in organizing biological sample collection and storage, conducting immunoassay testing, and supervising laboratory technicians in immunological techniques. She is a co-investigator in the Sacramento Area Latino Study of Aging (SALSA) and has supervised the immunoassay testing of antibodies to Cytomegalovirus, Herpes Simplex Virus Type-1, Herpes Simplex Virus Type-2, and C-reactive protein in over 1,000 frozen serum samples from the SALSA study.

TM is Associate Professor of Anthropology, Director of the Laboratory for Human Biology Research, and Associate Director of Cells to Society (C2S): Center for Social Disparities and Health at Northwestern University. His work is primarily concerned with the dynamic interrelationships among biology, culture, and individual psychosocial environments, with an emphasis on stress and the ecology of immune function. He has been a leader in the development and application of minimally-invasive methods for integrating physiological measures into population-based research, and is a leading expert in development of dried-blood spot assays for population research.

## Authors' contributions

JD conceived of the study, participated in its design and coordination, performed the immunoassays, performed the statistical analysis, and drafted the manuscript. AE conceived of the study, participated in its design and coordination, and critically revised the manuscript. LC carried out the immunoassays, performed the statistical analysis, and critically revised the manuscript. YH carried out the immunoassays and critically revised the manuscript. TM conceived of the study, participated in its design and coordination, oversaw the and critically revised the manuscript. All authors read and approved the final manuscript.
